# Atherogenic Index of Plasma (AIP): The Most Accurate Indicator of Overweight and Obesity Among Lipid Indices in Type 2 Diabetes—Findings From a Cross‐Sectional Study

**DOI:** 10.1002/edm2.70007

**Published:** 2024-11-05

**Authors:** Sahar Karimpour Reyhan, Amirhossein Yadegar, Sahar Samimi, Pooria Nakhaei, Alireza Esteghamati, Manouchehr Nakhjavani, Soheil Karimpour Reihan, Soghra Rabizadeh

**Affiliations:** ^1^ Endocrinology and Metabolism Research Center (EMRC), Vali‐Asr Hospital Tehran University of Medical Sciences Tehran Iran; ^2^ Mechanical Engineering School University of Tehran Tehran Iran

**Keywords:** atherogenic index of plasma, diabetes, lipid indices, obesity, overweight

## Abstract

**Background:**

This study aimed to evaluate the relationships of conventional and calculated lipid indices with obesity or overweight in patients with type 2 diabetes (T2D).

**Methods:**

In this cross‐sectional study, all participants were categorised into three groups: normal weight (18 ≤ BMI < 25), overweight (25 ≤ BMI < 30) and obese (BMI ≥ 30). Conventional lipid indices, including triglyceride (TG), total cholesterol, LDL‐C and HDL‐C, were measured. Lipid indices, including the atherogenic index of plasma (AIP), non‐HDL‐C, TC/HDL‐C, LDL‐C/HDL‐C, non‐HLD‐C/HDL‐C (atherogenic index, AI) and lipoprotein combine index (LCI), were calculated. The associations of these lipid indices with obesity and overweight status were evaluated using univariate and multivariate multinomial logistic regression analyses.

**Results:**

In this study, 2661 patients with T2D, including 651 patients with normal weight, 1144 with overweight, and 866 with obesity, were recruited. According to a multinomial logistic regression analysis after adjustment for multiple confounders, the odds ratio (OR) was greater for the AIP than for other conventional and calculated lipid indices in overweight and obese patients with T2D. The AIP had a significant relationship with overweight, with an OR of 4.79 (95% CI: 1.65–13.85), and it had a significant relationship with obesity, with an OR of 13.64 (95% CI: 3.96–47.04). According to the ROC curve, with a cut‐off value of 0.68, AIP could predict obesity with 82% sensitivity and 55% specificity (AUC = 0.770, 95% CI: 0.729–0.812, *p* < 0.001).

**Conclusion:**

Compared with other conventional and calculated lipid markers, the AIP is most strongly associated with obesity and overweight in patients with T2D.

## Graphical abstract

This cross‐sectional study showed that compared with other conventional and calculated lipid markers, the atherogenic index of plasma (AIP) is most strongly associated with obesity and overweight in patients with type 2 diabetes (T2D).

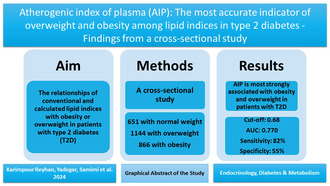

## 1 Introduction

The prevalence of diabetes is increasing steadily, from 108 million patients in 1980 to 463 million in 2019, with projections indicating a rise to 700 million by 2045 [1]. Concurrently, the global rise in obesity and overweight—conditions closely linked to sedentary lifestyles and poor dietary habits—has intensified public health concerns, particularly due to their role in promoting insulin resistance, the primary mechanism underlying type 2 diabetes (T2D) [2]. In clinical settings, obesity complicates the management of T2D and exacerbates associated comorbidities. Therefore, obesity is of significant importance both in clinical practice and in research settings [3]. In Iran, around 48.8% of patients living with diabetes were estimated to be obese [4]. While body mass index (BMI) is widely used, it often fails to account for the complexities of obesity‐related complications, necessitating more precise biomarkers [5]. The introduction of new indices, such as the atherogenic index of plasma (AIP), non‐high‐density lipoprotein cholesterol (non‐HDL‐C), total cholesterol/high‐density lipoprotein cholesterol (TC/HDL‐C), low‐density lipoprotein cholesterol (LDL‐C)/HDL‐C, atherogenic index (AI) and lipoprotein combine index (LCI), may be helpful in predicting obesity [6]. TG, abundant in adipose tissue, can cause lipotoxicity and insulin resistance [7]. HDL‐C, rich in lipids and proteins, has anti‐oxidant and anti‐inflammatory roles in diabetes [7]. The AIP, which reflects the ratio of TG to HDL‐C and lipoprotein particle size, offers a simple and more comprehensive assessment of dyslipidemia compared to individual lipid levels [7]. AIP was validated to be significantly correlated with diabetes, cardiovascular disease (CVD), and obesity [7]. However, the association between AIP and other lipid indices with overweight or obesity in patients with T2D remains inadequately explored. Understanding these associations could be instrumental in developing preventative measures and innovative management strategies for T2D. This study aimed to assess and compare the associations and effectiveness of various conventional and calculated lipid indices in predicting obesity and overweight in individuals with T2D.

## 2 Method and Material

### 2.1 Study Design and Population

This was a cross‐sectional study of an ongoing regional cohort of patients with T2D who were referred to the diabetes clinic of Vali‐Asr Hospital, which is affiliated with Tehran University of Medical Sciences, over 10 years from 2013 until 2022. Diabetes was diagnosed according to the American Diabetes Association (ADA) criteria [8]. The exclusion criteria were age < 30 years, cancer, hypothyroidism, hyperthyroidism, familial hypercholesterolemia, haemoglobinopathy, end‐stage renal disease (ESRD), dialysis, liver disease, oestrogen use by women and pregnancy. This research was approved by the research ethics committee of Tehran University of Medical Sciences with (Approval number: IR.TUMS.IKHC.REC.1399.426). Written informed consent was obtained from all study participants. This study was conducted in accordance with the Declaration of Helsinki.

### 2.2 Data Collection

Patient characteristics, including age, gender, duration of diabetes and medications in use were determined using interview by trained examiners. Qualified nurses performed anthropometric measurements, which included height, weight and waist circumference (WC). Height was assessed by an inflexible measurement tape with a precision of 0.1 cm. Weight was measured by a digital scale (Tefal PP1100, France) with a precision of 0.1 kg, while the patients were wearing light clothing. WC was measured at the midpoint between the lower borders of the rib cage and the iliac crest, rounded to the nearest 0.1 cm. BMI was determined by weight (kg) proportional to the square of the height (m^2^). Systolic and diastolic blood pressure measurements (SBP and DBP) were obtained with the participants in the seated position after 10 min of rest using calibrated sphygmomanometers (Omron M7 digital, Japan). The test was repeated after 15 min, and the average of two records was recorded. Blood pressure ≥ 140/90 mmHg or using medication to manage high blood pressure was classified as hypertension [9]. The estimated glomerular filtration rate (eGFR) was estimated using the Cockroft‐Gault equation for each participant ([140 − age in years] × weight in kg)/(serum creatinine in mg/dL × 72, in women multiplied by 0.85).

After 12 h of overnight fasting, laboratory measurements were performed. HbA1c was tested by high‐performance liquid chromatography (A1C, DS5 Pink kit; Drew, France). Fasting blood sugar (FBS), 2‐h postprandial blood glucose (2hpp), creatinine and serum lipid profile (TG, TC, HDL‐C, LDL‐C) were measured using enzymatic methods by a Technicon RA‐analyser (Pars Azmon, Karaj, Iran).

The atherogenic index of plasma (AIP) was computed as Log (TG/HDL‐C). Non‐HDL‐C was calculated as TC minus HDL‐C. The atherogenic index (AI) was calculated as non‐HDL‐C/HDL‐C. The LCI was calculated as TC*TG*LDL‐C/HDL‐C. These lipid indices were compared among three groups of patients according to their BMI, namely, normal weight (18 ≤ BMI < 25), overweight (25 ≤ BMI < 30) and obese (BMI ≥ 30) [10].

### 2.3 Statistical Analysis

Analysis was performed using SPSS version 22 (IBM Corporation, New York, USA) and Python version 3.12 with NumPy version 1.26, Panda's version 2.1.4, Matplotlib version 3.8.1 and Scikit‐learn version 1.4.0 libraries [11, 12, 13, 14]. This analysis used probability graphics and the Kolmogorov–Smirnov test to assess whether the variables followed a normal distribution. Continuous variables were presented as mean ± standard deviation (SD), and categorical variables were presented as frequencies (percentages). One‐way Analysis of Variance (ANOVA) was used to compare quantitative variables between groups, followed by Tukey's Post Hoc test. The chi‐square test was applied to compare categorical variables. Variables with non‐normal distribution were presented as median (Q1, Q3), and the median between groups was compared using Kruskal–Wallis test. Univariate multinomial logistic regression was used to evaluate the relationship between lipid indices and overweight and obesity. Furthermore, multinomial logistic regression was utilised to assess these relationships after adjustment for confounding variables in three models. Model 1 was adjusted for age, sex, duration of diabetes and hypertension. Model 2 was adjusted for age, sex, duration of diabetes, hypertension, eGFR, HbA1c, SBP and DBP. Model 3 was adjusted for age, sex, duration of diabetes, hypertension, eGFR, HbA1c, SBP, DBP, diabetes drugs and anti‐lipid drugs. Moreover, this analysis utilised log LCI due to the non‐normal distribution of LCI. A receiver operating characteristic (ROC) curve was employed to assess the predictive efficacy of AIP for the obese group, and the threshold for AIP was determined by utilising the maximum Youden Index. A *p* < 0.05 indicated statistical significance.

## 3 Results

### 3.1 Baseline Characteristics

A total of 2661 patients with T2D, including 1595 (59.9%) females and 1067 (40.1%) males, have an average age of 57.3 ± 10.9 years. All participants were categorised into three groups according to their BMI; 24.5% were normal weight, 43.0% were overweight, and 32.5% were obese.

As shown in Table 1, the median of AIP in the obese and overweight groups was notably greater than in the normal weight group (*p* < 0.001). In addition, AIP levels were significantly higher in the obese group compared to the overweight group (*p* < 0.001).

**TABLE 1:** Baseline characteristics of the study population based on BMI categories.

Variables		BMI (kg/m^2^)			*p*
		< 25 Normal weight (*n* = 651)	25–30 Overweight (*n* = 1144)	> 30 Obese (*n* = 866)	
Age (years)		58.5 ± 11.7	57.5 ± 10.6	56.0 ± 10.2* ^,^ ^#^	< 0.001
Gender (women to men)		295/356	665/480	635/231	< 0.001
Duration of diabetes (years)		8 (3–13.25)	8 (3–12)	6 (3–11)* ^,^ ^#^	0.002
WC (cm)		86.5 ± 8.4	94.5 ± 7.8*	105.7 ± 10.8* ^,^ ^#^	< 0.001
Hypertension (*n*, %)		262 (39.3%)	621 (53.0%)	529 (60.2%)	< 0.001
SBP (mmHg)		126.3 ± 19.6	131.8 ± 20.5*	136.3 ± 20.7* ^,^ ^#^	< 0.001
DBP (mmHg)		75.7 ± 11.6	78.6 ± 11.9*	81.4 ± 12.9* ^,^ ^#^	< 0.001
FBS (mg/dL)		192.7 ± 86.2	179.8 ± 72.7*	177.3 ± 64.6*	< 0.001
2hpp (mg/dL)		263.6 ± 110.9	245.0 ± 101.2*	237.9 ± 92.6*	< 0.001
HbA1c (%)		8.40 ± 2.07	8.20 ± 1.87	8.09 ± 1.72*	0.014
TC (mg/dL)		176.9 ± 47.3	177.1 ± 46.7	177.9 ± 45.2	0.920
TG (mg/dL)		125 (91.5–185)	143.5 (105–210)*	160 (120–229)* ^,^ ^#^	< 0.001
LDL‐C (mg/dL)		99.7 ± 36.3	100.7 ± 35.3	98.8 ± 34.1	0.535
HDL‐C (mg/dL)		44.4 ± 11.2	43.0 ± 9.8	43.4 ± 10.3	0.065
Cr (mg/dL)		1.03 ± 0.28	1.02 ± 0.34	0.99 ± 0.30	0.205
eGFR (mL/min/1.73m^2^)		67.3 ± 21.7	80.6 ± 25.5*	97.1 ± 31.0* ^,^ ^#^	< 0.001
**Lipid indices**					
AIP		0.45 (0.29–0.66)	0.54 (0.38–0.73)*	0.58 (0.43–0.74)* ^,^ ^#^	< 0.001
Non‐HDL‐C (mg/dL)		131.2 ± 45.6	133.7 ± 45.4	133.3 ± 43.2	0.585
TC/HDL‐C		4.16 ± 1.43	4.26 ± 1.33	4.26 ± 1.46	0.378
LDL‐C/HDL‐C		2.35 ± 0.97	2.42 ± 0.94	2.37 ± 0.95	0.343
AI		3.16 ± 1.43	3.26 ± 1.33	3.26 ± 1.46	0.378
Log LCI		4.68 ± 0.43	4.76 ± 0.41*	4.79 ± 0.38*	< 0.001
**Medication**					
Antihyperglycemic agents (*n*, %)	OAD	475 (73.0%)	827 (72.3%)	586 (67.7%)	< 0.001
	Insulin	94 (14.4%)	141 (12.3%)	88 (10.2%)	
	OAD + Insulin	82 (12.6%)	176 (15.4%)	192 (22.1%)	
Dyslipidemia drug (*n*, %)	Statins	636 (97.7%)	1120 (97.9%)	846 (97.7%)	0.936
	Fibrates	15 (2.3%)	24 (2.1%)	20 (2.3%)	

*Note:* Data are presented as mean ± standard deviation (SD) or median (Q1 − Q3).

Abbreviations: 2hpp: two‐hour postprandial plasma glucose; AI: atherogenic index; AIP: atherogenic index of plasma; BMI: body mass index; Cr: creatinine; DBP: diastolic blood pressure; eGFR: estimated glomerular filtration rate; FBS: fasting blood sugar; HbA1c: haemoglobin A1c; HDL‐C: high‐density lipoprotein cholesterol; LCI: lipoprotein combine index; LDL‐C: low‐density lipoprotein cholesterol; non‐HDL‐C: non‐high‐density lipoprotein cholesterol; OAD: oral antidiabetic drug; SBP: systolic blood pressure; TC: total cholesterol; TG: triglycerides; WC: waist circumference.

* Significant difference compared to normal BMI group.

^#^ Significant difference between overweight and obese groups.

Patients with T2D and obesity had higher WC, SBP, DBP, TG and eGFR levels than patients with normal weight (*p* < 0.05). Moreover, overweight patients had significantly greater WC, SBP, DBP, TG and eGFR and lower FBS and 2hpp levels than patients in the normal weight group (*p* < 0.05). Patients with obesity had lower age, duration of diabetes, FBS, 2hpp and HbA1c levels than did normal weight participants (*p* < 0.05). The results also showed no significant difference in HDL‐c among the three groups. Patients with obesity had higher WC, SBP, DBP, TG and eGFR levels and lower age and duration of diabetes in comparison with the overweight group (Table 1).

### 3.2 Association Between Lipid Indices and Overweight and Obesity in Patients With T2D

According to Table 2, univariate logistic regression analyses of lipid parameters in the overweight and obese groups revealed an odds ratio of 2.9 for the AIP in the overweight group compared with that in the normal weight group (CI: 1.92–4.41, *p* < 0.001) and an odds ratio of 4.3 in the obese group compared with that in the normal weight group (CI: 2.8–6.7, *p* < 0.001). Additionally, compared with those of normal‐weight patients, triglyceride levels had an odds ratio of 1.002 for both overweight and obese patients (CI: 1.001–1.003, *p* < 0.001 and CI: 1.001–1.004, *p* < 0.001, respectively). There was an odds ratio of 0.98 for HDL cholesterol in the overweight group compared to the normal‐weight group (CI: 0.977–0.998, *p* = 0.017). Other conventional or calculated lipids had no significant association with overweight or obesity, except for the LCI. Due to the non‐normal distribution, the logarithmic value of the LCI was included in the logistic regression analysis, and the results showed an odds ratio of 1.64 (CI: 1.21–2.22, *p* < 0.001) in overweight patients compared to normal‐weight participants with T2D and an odds ratio of 2.1 (CI: 1.59–3.02, *p* < 0.001) in patients with obesity compared to normal‐weight participants with T2D.

**TABLE 2:** Univariate multinomial logistic regression analysis of lipid parameters comparing overweight and obesity status to normal weight.

Variables	Overweight		Obese	
	OR^a^	95% CI	OR^a^	95% CI
**Traditional lipids**				
TG (mg/dL)	1.002	1.001–1.003	1.002	1.001–1.004
TC (mg/dL)	1	0.998–1.002	1	0.998–1.003
HDL‐C (mg/dL)	0.987	0.977–0.998	0.991	0.980–1.002
LDL‐C (mg/dL)	1.001	0.998–1.004	0.999	0.996–1003
**Calculated lipid indices**				
AIP	2.9	1.92–4.41	4.3	2.8–6.7
Non‐HDL‐C (mg/dL)	1.001	0.999–1.004	1.001	0.999–1.004
Total Chol/HDL‐C	1.054	0.972–1.143	1.054	0.969–1.147
LDL‐C/HDL‐C	1.81	0.962–1.214	1.023	0.905–1.157
AI	1.054	0.972–1.143	1.054	0.972–1.143
Log LCI	1.64	1.21–2.22	2.1	1.59–3.02

Abbreviations: AI: atherogenic index; AIP: atherogenic index of plasma; CI: confidence interval; HDL‐C: high‐density lipoprotein cholesterol; LCI: lipoprotein combination index; LDL‐C: low‐density lipoprotein cholesterol; non‐HDL‐C: non‐high‐density lipoprotein cholesterol; OR: odds ratio; TC: total cholesterol; TG: triglycerides.

^a^ Normal weight was used as reference.

The results of the multivariable logistic regression analysis are presented in Table 3 and Table 4. AIP had an odds ratio of 2.98 (CI: 1.95–4.54, *p* < 0.001) in Model 1, 3.43 (CI: 2.13–5.53, *p* < 0.001) in Model 2 and 4.79 (CI: 1.65–13.85, *p* = 0.004) in Model 3 in the overweight group compared to the normal‐weight group.

**TABLE 3:** Multivariable multinomial logistic regression analysis of lipid parameters for the overweight group.

Lipids	Model 1		Model 2		Model 3	
	OR	95% CI	OR	95% CI	OR	95% CI
**Traditional lipids**						
TG (mg/dL)	1.002	1.001–1.003	1.002	1.001–1.003	1.003	1.000–1.006
TC (mg/dL)	1.000	0.998–1.003	1.000	0.997–1.003	1.000	0.995–1.005
HDL‐C (mg/dL)	0.989	0.980–0.998	0.987	0.978–0.997	0.979	0.957–1.001
LDL‐C (mg/dL)	1.001	0.998–1.004	1.000	0.997–1.004	0.999	0.992–1.006
**Calculated lipid indices**						
AIP	2.985	1.959–4.548	3.439	2.139–5.530	4.79	1.65–13.85
Non‐HDL‐C (mg/dL)	1.002	0.999–1.004	1.001	0.998–1.004	1.001	0.996–1.007
Total Chol/HDL‐C	1.092	1.004–1.188	1.099	0.999–1.209	1.206	0.957–1.519
LDL‐C/HDL‐C	1.125	0.999–1.268	1.110	0.971–1.269	1.148	0.843–1.563
AI	1.082	0.99–1.17	1.083	0.99–1.02	1.03	0.72–1.48
Log LCI	1.70	1.24–2.32	1.73	1.21–2.45	1.24	0.40–3.76

*Note:* Model 1: adjusted for age, sex, duration of diabetes and hypertension. Model 2: adjusted for age, sex, duration of diabetes, hypertension, eGFR, HbA1c, systolic blood pressure and diastolic blood pressure. Model 3: adjusted for age, sex, duration of diabetes, hypertension, eGFR, HbA1c, systolic blood pressure, diastolic blood pressure, diabetes drugs and anti‐lipid drugs.

Abbreviations: AI: atherogenic index; AIP: atherogenic index of plasma; CAD: coronary artery disease; CI: confidence interval; eGFR: estimated glomerular filtration rate; HbA1c: haemoglobin A1c; HDL‐C: high‐density lipoprotein cholesterol; LCI: lipoprotein combination index; LDL‐C: low‐density lipoprotein cholesterol; non‐HDL‐C: non‐high‐density lipoprotein cholesterol; OR: odds ratio; TC: total cholesterol; TG: triglycerides.

**TABLE 4:** Multivariable multinominal logistic regression analysis of lipid parameters for the obese group.

Lipids	Model 1		Model 2		Model 3	
	OR	95% CI	OR	95% CI	OR	95% CI
**Traditional lipids**						
TG	1.002	1.001–1.003	1.002	1.001–1.004	1.005	1.001–1.008
Chol	1.000	0.997–1.003	0.998	0.995–1.001	0.999	0.993–1.005
HDL‐C	0.987	0.977–0.996	0.985	0.974–0.997	0.970	0.945–0.995
LDL‐C	0.999	0.995–1.002	0.996	0.992–1.001	0.994	0.986–1.003
**Calculated lipid indices**						
AIP	4.339	2.732–6.89	5.097	2.897–8.967	13.649	3.96–47.04
Non‐HDL‐C	1.001	0.998–1.004	0.999	0.995–1.002	1.001	0.994–1.007
Total Chol/HDL‐C	1.098	1.002–1.202	1.077	0.961–1.207	1.232	0.951–1.596
LDL‐C/HDL‐C	1.082	0.950–1.233	1.025	0.874–1.204	1.036	0.731–1.468
AI	1.09	0.99–1.19	1.06	0.95–1.18	1.24	0.81–1.88
Log LCI	2.13	1.51–3.00	1.82	1.20–2.76	3.36	0.896–12.46

*Note:* Model 1: adjusted for age, sex, duration of diabetes and hypertension. Model 2: adjusted for age, sex, duration of diabetes, hypertension, eGFR, HbA1c, systolic blood pressure and diastolic blood pressure. Model 3: adjusted for age, sex, duration of diabetes, hypertension, eGFR, HbA1c, systolic blood pressure, diastolic blood pressure, diabetes drugs and anti‐lipid drugs.

Abbreviations: AI: atherogenic index; AIP: atherogenic index of plasma; CAD: coronary artery disease; CI: confidence interval; eGFR: estimated glomerular filtration rate; HbA1c: haemoglobin A1c; HDL‐C: high‐density lipoprotein cholesterol; LCI: lipoprotein combination index; LDL‐C: low‐density lipoprotein cholesterol; non‐HDL‐C: non‐high‐density lipoprotein cholesterol; OR: odds ratio; TC: total cholesterol; TG: triglycerides.

Additionally, odds ratios of 4.33 (CI: 2.73–6.89, *p* < 0.001) in Model 1, 5.097 (CI: 2.89–8.96, *p* < 0.001) in Model 2 and 13.64 (CI: 3.96–47.04, *p* < 0.001) in Model 3 were detected in patients with obesity compared with those in the normal‐weight group.

The total cholesterol/HDL cholesterol ratio showed odds ratios of 1.092 (CI: 1.004–1.188, *p* = 0.04) in Model 1, 1.099 (CI: 0.999–1.209, *p* = 0.052) in Model 2, 1.206 (CI: 0.957–1.519, *p* = 0.11) in Model 3 and 1.098 (CI: 1.002–1.202, *p* = 0.04) in Model 1; 1.077 (CI: 0.961–1.207, *p* = 0.20) in Model 2; and 1.232 (CI: 0.951–1.596, *p* = 0.11) in Model 3 in patients with obesity in comparison with the normal‐weight group.

Additionally, the Log LCI (lipoprotein combination index) had an odds ratio of 1.70 (CI: 1.24–2.32, *p* = 0.001) in Model 1, 1.73 (CI: 1.21–2.45, *p* = 0.002) in Model 2 and 1.24 (CI: 0.40–13.76, *p* = 0.70) in Model 3, in the overweight group, compared to the normal‐weight group; and an odds ratio of 2.13 (CI: 1.51–3.00, *p* = 0.001) in Model 1, 1.82 (CI: 1.20–2.76, *p* = 0.005) in Model 2 and 3.36 (CI: 0.896–12.46, *p* = 0.07) in Model 3, in patients with obesity in comparison with the normal‐weight group. There were no significant differences in the odds ratios of other indices between the overweight and obese groups and the normal‐weight group (Tables 3 and 4).

### 3.3 ROC Curve Analysis

**FIGURE 1:**
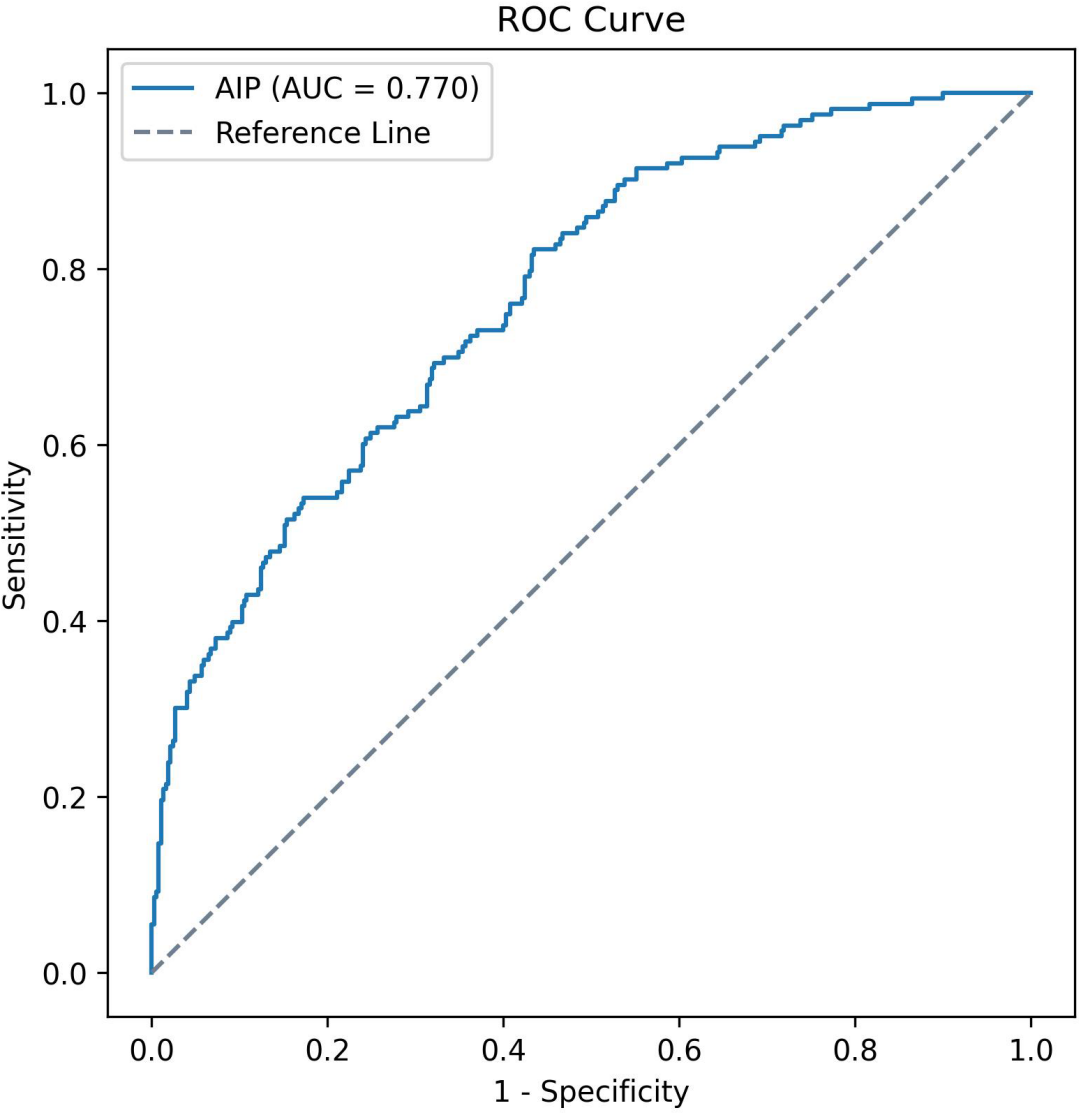
ROC curve, sensitivity and specificity of AIP in diagnosis of obesity in patients with T2D; adjusted for age, sex, duration of diabetes, hypertension, eGFR, HbA1c, systolic blood pressure, diastolic blood pressure, diabetes drugs and anti‐lipid drugs. AIP: Atherogenic index of plasma; eGFR: Estimated glomerular filtration rate; HbA1c: Haemoglobin A1; ROC: Receiver operating characteristic; T2D: Type 2 diabetes.

Using the maximum Youden Index, with a cut‐off value of 0.68, AIP could predict obesity with 82% sensitivity and 55% specificity (AUC = 0.770, 95% CI: 0.729–0.812, *p* < 0.001) (Figure 1).

## 4 Discussion

This study showed that obesity and overweight status were strongly correlated with higher AIP in patients with T2D. The findings revealed that among conventional and calculated lipid biomarkers, including the AIP, non‐HDL‐C, TC/HDL‐C, LDL‐C/HDL‐C, atherogenic index and LCI, the AIP had the most independent association with both obesity and overweight in patients with T2D after adjustment for age, sex, duration of diabetes, hypertension, eGFR, HbA1c, SBP, DBP, diabetes drugs, and anti‐lipid drugs.

Considering that the GFR rate was significantly higher in overweight and obesity groups with greater superiority in the obesity group; we had done adjustment in regression analysis.

Patients with T2D have mild hypertriglyceridemia often due overproduction of TG‐rich lipoproteins in the liver, associated with decreased high‐density lipoprotein (HDL) cholesterol level as a result of defective LPL catabolism of TG‐rich lipoproteins. Moreover, small dense low‐density lipoproteins (LDL) and apolipoprotein B levels are increased in these patients by cholesterol ester transfer protein activity. Overweight and obesity are associated with more insulin resistance, hypertension and worsening of above‐mentioned lipoprotein abnormalities in patients with T2D [15].

The AIP, the logarithmic ratio of triglyceride to HDL, is a plasma atherogenic marker that is significantly greater in atherosclerosis‐related CVD. This index seems to be more closely related to the risk of CVD and mortality than other atherogenic indices or lipoprotein concentrations alone [16]. AIP was reported as a novel independent prognostic biomarker of coronary artery disease beyond traditional risk factors in T2D [17]. Previous studies suggest that high triglyceride and low HDL levels play a substantial role in the development and progression of insulin resistance. Higher TG levels lead to lipotoxicity which can contribute to the progression of insulin resistancy in T2D. HDL‐c has several anti‐oxidant and anti‐inflammatory functions and lower HDL values decrease cholesterol efflux and rises cholesterol accumulation in pancreatic beta cells affecting their function [18]. AIP levels not only account for triglyceride to HDL level in T2D, but also represent the size of lipoprotein particles which reflects the pathogenicity and characterises dyslipidemia better than high triglyceride or low HDL levels, and it can determine the level of abnormal lipid metabolism and the severity of insulin resistance in T2D [7].

In a study by Frohlich and Dobiasova [19] the AIP was a superior predictor of CAD compared to conventional lipid markers. Furthermore, the AIP is positively correlated with the incidence of micro‐ and macrovascular complications of T2D [6, 20, 21, 22, 23, 24]. In patients with T2D, as shown in a study by Li et al. [20], there is a link between AIP and chronic microvascular complications of diabetes. Additionally, they found a correlation between higher AIP values and metabolic syndrome in patients with T2D.

In this study, after combining TG and HDL to create the AIP, the odds ratio dramatically increased, suggesting that the AIP is a much better index related to increased BMI in patients with T2D than TG or HDL alone. Previous studies on the correlation between the AIP and obesity were mostly performed in patients without diabetes, and a positive correlation was found between an increase in BMI and the AIP. For example, in a study by Niroumand et al. in 2014, it was shown that the AIP was a predictor of cardiovascular events in the normal population and was directly associated with obesity, waist circumference and decreased physical activity. The mean AIP in their study of the general population was 0.41 for male subjects and 0.35 for female subjects [25]. In our investigation, the median of AIP was 0.45, 0.54 and 0.58 in the normal weight, overweight and obese patients with T2D, respectively. Additionally, in a study in India, the atherogenic index was used as a predictor of cardiovascular risk among middle‐aged women in four groups with different BMIs in the normal population and revealed that an increased BMI was correlated with an increase in the plasma atherosclerotic index [26]. Another study by Xiaowei Zhu et al. in China examined 6465 people from the general population, 503 of whom were obese (BMI > 28). They reported that increased AIP had a strong positive association with higher BMI and LDL‐C [27].

In patients living with obesity and diabetes, several studies have shown correlations between obesity and elevated levels of total cholesterol, LDL and TG and decreased levels of HDL [28], and correlations between lipid atherogenic indices and metabolic‐associated fatty liver disease and insulin resistance have also been reported in previous studies [29, 30]. However, they did not compare TC/HDL‐C, LDL‐C/HDL‐C, the AI or the LCI with obesity in patients living with T2D. Moreover, our results showed that this strong association was also present in overweight patients with T2D.

T2D on its own is a major risk factor for CVD, and obesity usually predisposes individuals to hypertension and dyslipidemia, which are also major risk factors for CVD; therefore, it is not surprising that obese patients with T2D are at a greater risk of CVD [31, 32, 33, 34]. With a 20%–30% increase in body weight, the mortality rate increased by 2.5‐ to 3.3‐fold. The mortality rate is even greater when body weight increases to more than 40% of the ideal weight by 5.2–7.9 times [35]. The aforementioned evidence emphasises the significance of monitoring obesity in patients with T2D.

Some limitations in this study were as follows. First, the cross‐sectional nature of this survey limits the ability to deduce a causal relationship between conventional and calculated lipid markers and BMI status. Second, lipid indices were measured at a single visit, and variations in lipid serum levels should be considered in future research. Third, although this survey took place in a tertiary referral hospital, future multicenter‐based research will increase the reliability of the results.

## 5 Conclusion

This study revealed that compared with other conventional and calculated lipid markers, the AIP is strongly related to not only obesity but also overweight in patients with T2D. Therefore, the AIP, which is a simple and valuable biomarker of T2D, may be useful in clinical practice and epidemiological studies.

## Author Contributions

**Sahar Karimpour Reyhan:** investigation, data curation, methodology, writing – original draft. **Amirhossein Yadegar:** formal analysis, software, writing – review and editing. **Sahar Samimi:** visualization. **Pooria Nakhaei:** writing – review and editing. **Alireza Esteghamati:** supervision, writing – review and editing. **Manouchehr Nakhjavani:** supervision, writing – review and editing, conceptualization. **Soheil Karimpour Reihan:** writing – review and editing. **Soghra Rabizadeh:** validation, formal analysis, project administration, conceptualization, writing – review and editing.

## Ethics Statement

This study was ethically approved by Tehran University of Medical Sciences (Approval number: IR.TUMS.IKHC.REC.1399.426) and was performed in accordance with the ethical standards of the 1964 Declaration of Helsinki.

## Consent

All patients in this study provided written consent.

## Conflicts of Interest

The authors declare no conflicts of interest.

## Data Availability

The data that support the findings of this study are available from the corresponding author upon reasonable request.
